# Rare earth elements induce cytoskeleton-dependent and PI4P-associated rearrangement of SYT1/SYT5 endoplasmic reticulum–plasma membrane contact site complexes in Arabidopsis

**DOI:** 10.1093/jxb/eraa138

**Published:** 2020-03-17

**Authors:** EunKyoung Lee, Brenda Vila Nova Santana, Elizabeth Samuels, Francisco Benitez-Fuente, Erica Corsi, Miguel A Botella, Jessica Perez-Sancho, Steffen Vanneste, Jiří Friml, Alberto Macho, Aristea Alves Azevedo, Abel Rosado

**Affiliations:** 1 Department of Botany, University of British Columbia, Vancouver, Canada; 2 Departamento de Biologia Vegetal, Universidade Federal de Viçosa, Viçosa, Minas Gerais, Brazil; 3 Instituto de Hortofruticultura Subtropical y Mediterránea, Universidad de Málaga–Consejo Superior de Investigaciones Científicas (IHSM-UMA-CSIC), Departamento de Biología Molecular y Bioquímica, Universidad de Málaga, Málaga, Spain; 4 Shanghai Center for Plant Stress Biology, CAS Center for Excellence in Molecular Plant Sciences, Shanghai Institute of Biological Sciences, Chinese Academy of Sciences, Shanghai, China; 5 Department of Plant Biotechnology and Bioinformatics, Ghent University, Ghent, Belgium; 6 Center for Plant Systems Biology, VIB, Ghent, Belgium; 7 Ghent University Global Campus, Incheon, Korea; 8 Institute of Science and Technology (IST), Klosterneuburg, Austria; 9 University of Essex, UK

**Keywords:** Arabidopsis, calcium, cytoskeleton, endoplasmic reticulum (ER), ER–PM membrane contact sites, phosphoinositides, PI4P, plasma membrane (PM), rare earth elements, stress adaptation, synaptotagmins, SYT1/SYT5

## Abstract

In plant cells, environmental stressors promote changes in connectivity between the cortical endoplasmic reticulum (ER) and the plasma membrane (PM). Although this process is tightly regulated in space and time, the molecular signals and structural components mediating these changes in interorganelle communication are only starting to be characterized. In this report, we confirm the presence of a putative tethering complex containing the synaptotagmins 1 and 5 (SYT1 and SYT5) and the Ca^2+^- and lipid-binding protein 1 (CLB1/SYT7). This complex is enriched at ER–PM contact sites (EPCSs), has slow responses to changes in extracellular Ca^2+^, and displays severe cytoskeleton-dependent rearrangements in response to the trivalent lanthanum (La^3+^) and gadolinium (Gd^3+^) rare earth elements (REEs). Although REEs are generally used as non-selective cation channel blockers at the PM, here we show that the slow internalization of REEs into the cytosol underlies the activation of the Ca^2+^/calmodulin intracellular signaling, the accumulation of phosphatidylinositol-4-phosphate (PI4P) at the PM, and the cytoskeleton-dependent rearrangement of the SYT1/SYT5 EPCS complexes. We propose that the observed EPCS rearrangements act as a slow adaptive response to sustained stress conditions, and that this process involves the accumulation of stress-specific phosphoinositide species at the PM.

## Teaser

Rare earth elements induce ER membrane remodeling and increase ER–PM connectivity in a process that involves phosphoinositide-associated reorganization of synaptotagmin-tethering complexes.

## Introduction

A hallmark of eukaryotic cells is the establishment of physical interfaces that enable organelle to organelle direct communication. These interfaces, known as membrane contact sites (MCSs), serve as platforms for the control of essential cellular functions including metabolism, regulation of organelle dynamics, and stress signal integration (Helle *et al.*, 2013; Bravo-Sagua *et al.*, 2014; Prinz, 2014; Pérez-Sancho *et al*., 2016a). In plants, the establishment of MCSs between the endoplasmic reticulum (ER) and different organelles is particularly important for the coordination of key physiological functions including lipid transfer (e.g. ER–mitochondria and ER–plastid contact sites; Xu *et al.*, 2008; Block and Jouhet, 2015; Fan *et al.*, 2015; Michaud *et al.*, 2016), intercellular communication (e.g. plasmodesmata; Uchiyama *et al.*, 2014; Levy *et al.*, 2015; Tilsner *et al.*, 2016; Ishikawa *et al.*, 2020), organelle distribution (e.g. ER–peroxisome, ER–plastid, and ER–Golgi contact sites; Andersson *et al.*, 2007; Sparkes *et al.*, 2009; Barton *et al.*, 2013), and the Ca^2+^-dependent regulation of stress responses [ER–plasma membrane (PM) contact sites (EPCSs); Schapire *et al.*, 2008; Yamazaki *et al.*, 2008; P. Wang et al., 2014, 2016; Pérez-Sancho *et al.*, 2015; Kim *et al.*, 2016; Lee *et al.*, 2019).

In Arabidopsis, the dynamic arrangement of EPCSs is regulated by multiple families of EPCS components, namely synaptotagmins (SYTs), vesicle-associated membrane protein (VAMP)-associated proteins 27 (VAP27s), NETWORKED 3 (NET3C), and VAP-RELATED SUPPRESSORS OF TOO MANY MOUTHS (VSTs) (Pérez-Sancho *et al.*, 2015; Wang *et al.*, 2017). These EPCS components serve a number of well-characterized functions including the response to biotic and abiotic stressors (Schapire *et al.*, 2008; Yamazaki *et al.*, 2008; Uchiyama *et al.*, 2014; Lewis and Lazarovitch, 2015; Levy *et al.*, 2015; Pérez-Sancho *et al.*, 2015; Kim *et al.*, 2016; Lee *et al.*, 2019), the control of the interactions between the ER and the cortical cytoskeleton (P. Wang et al., 2014, 2016; Siao *et al.*, 2016), and the activation of signal transduction events through the activation of receptor-like kinases (Ho *et al.*, 2016).

This study expands on existing research on the Arabidopsis SYT1, which is an EPCS tether that localizes in immobile cortical ER tubules, and docks the PM through Ca^2+^-dependent interactions between its C2 domains and negatively charged phospholipids (Schapire *et al.*, 2008; Yamazaki *et al.*, 2010; Pérez-Sancho et al., 2015, 2016b; Ishikawa *et al.*, 2018). Genetic studies using *syt1* loss-of-function mutants have shown that SYT1 is required for the control of immune secretory pathways (Kim *et al.*, 2016), maintenance of the cortical ER stability (Siao *et al.*, 2016), the regulation of cell to cell communication (Lewis and Lazarowitz, 2010; Uchiyama *et al.*, 2014; Levy *et al.*, 2015; Ishikawa *et al.*, 2020), and the tolerance to ionic, mechanical, and freezing stresses (Schapire *et al.*, 2008; Yamazaki *et al.*, 2010; Pérez-Sancho *et al.*, 2015). To achieve such diverse functions, SYT1 establish interactions with elements of the exocytic soluble SNAREs (Kim *et al.*, 2016), phytosterol-binding proteins (Dalal *et al.*, 2016), and reticulon proteins (Kriechbaumer *et al.*, 2015).

Recent studies have partially elucidated SYT1’s mechanism of action by showing it increases ER–PM connectivity by promoting the cytoskeleton-independent and phosphatidylinositol 4,5-bisphosphate [PI(4,5)P_2_]-associated EPCS expansion (Lee *et al.*, 2019), and that SYT1-labeled ER tubules can be disrupted by pharmacologically decreasing the intracellular Ca^2+^ concentration (Ishikawa *et al.*, 2018). Despite these advances, many aspects including the specificity of the PI(4,5)P_2_ signal as a trigger for EPCS expansion, and the dynamics of EPCS organization in response to extracellular Ca^2+^ depletion remain largely unexplored.

In this study, we corroborate a recent report describing the establishment of a putative tethering complex between the synaptotagmins 1 and 5, and the Ca^2+^-dependent lipid binding protein CLB1/SYT7 (hereafter CLB1) at EPCSs (Ishikawa *et al.*, 2020), and we expand their analysis by showing that SYT1 and SYT5 can form homo- and heterodimers *in vivo*. We also show that changes in extracellular Ca^2+^ have a limited effect in EPCS organization with the exception of treatments with salts of the rare earth elements (REEs) lanthanum (La^3+^) and gadolinium (Gd^3+^). Short-term treatments with REEs (minutes) have been classically used to block non-selective cation channels (Biagi and Enyeart, 1990; Lansman, 1990; Elinder and Arhem, 1994) and/or stretch-activated Ca^2+^-permeable channels at the PM (Yang and Sachs, 1989; Franco *et al.*, 1991; Hamill and McBride, 1996; Ermakov *et al.*, 2010), but recent studies have shown that long-term treatments with REEs promote their internalization and activate endocytosis in plant cells (L. Wang et al., 2014, 2016, 2019). Here we show that the dynamics of the REE-induced EPCS reorganization are not consistent with the Ca^2+^ channel-blocking activity of REEs at the PM but rather is a consequence of their slow internalization to the cytosol. We also show that the EPCS-remodeling process is associated with the activation of the Ca^2+^ signaling in the cytosol, and the accumulation of phosphatidylinositol-4-phosphate (PI4P) at the PM.

Our results highlight commonalities between the EPCS remodeling triggered by REEs (this study) and NaCl (Lee *et al.*, 2019), such as the slow dynamics of the remodeling process and the concomitant accumulation of negatively charged phosphoinositides at the PM. These findings also uncover key differences such as the identity of the phosphoinositide species that are accumulated, PI4P for REEs (this study), and PI(4,5)P_2_ for NaCl (Lee *et al.*, 2019), and the differential requirement for a functional cortical cytoskeleton for REE- and NaCl-induced EPCS remodeling. In a broader context, our study shows that the direct manipulation of extracellular Ca^2+^ levels has limited effects on plant EPCS organization, and supports a model where the slow accumulation of stress-specific phosphoinositide species at the PM acts as a general adaptive mechanism governing cortical ER–PM communication during sustained stress conditions.

## Materials and methods

### Plant materials and growth conditions

*Arabidopsis thaliana* Columbia (Col-0) was used as the wild type and the background for transgenes. Seeds of the mutants *syt5-1* (SALK_036961) and *clb1-2* (SALK_006298) were obtained from the Arabidopsis Biological Resource Center (Ohio State University). Previously published lines in this study are SYT1–green fluorescent protein (GFP) and MAPPER–GFP (Lee *et al.*, 2019); GFP–HDEL (Batoko *et al.*, 2000); 35S::C2AB (Pérez-Sancho *et al.*, 2015); GCaMP3 (DeFalco *et al.*, 2017); CITRINE-2×PH^PLC^; and CITRINE 1×PH^FAPP^ (Simon *et al.*, 2016). Plants were grown on half-strength Murashige and Skoog (MS) medium (Caisson Labs) or soil (Sunshine mix #4, Sun Gro Horticulture Canada Ltd) at 22 °C with a 16 h light/8 h dark cycle. For the NaCl assays, Arabidopsis seedlings were grown vertically for 4 d on 1/10th strength MS medium, and similar sized seedlings were transferred to the same medium supplemented with different NaCl concentrations. The root elongation and root hair phenotypes were scored after 9 d.

### Co-immunoprecipitation and large-scale immunoprecipitation for LC-MS/MS

Large-scale immunoprecipitation (IP) assays for LC-MS/MS were performed as described before (Kadota *et al.*, 2016), using 5–8 g of 10-day-old Arabidopsis seedlings stably expressing p35S:GFP (Lee *et al.*, 2019; Y. Wang *et al.*, 2019). For targeted co-IPs, 1 g of 10-day-old Arabidopsis stable transgenic lines expressing p35S:GFP (control), pUB10:CLB1-GFP, and pSYT5:SYT5-GFP were used. Protein extraction buffer was (for both targeted and LC-MS/MS-coupled co-IP): 50 mM Tris–HCl pH 7.5, 150 mM NaCl, 10% glycerol, 10 mM EDTA, 1 mM Na_2_MoO_4_, 1 mM NaF, 0.5 mM Na_3_VO_4_, 10 mM DTT, 0.5 mM phenylmethylsulfonyl fluoride (PMSF), 1% (v/v) protease inhibitor cocktail, and 1% NP-40. Washing buffer was the same, but with only 0.2% NP-40. Total proteins were extracted by incubation with the extraction buffer for 40–50 min. IP was performed with GFP-Trap beads (Chromotek, Planegg-Martinsried, Germany). Proteins were stripped from the beads by boiling in 50 μl of SDS loading buffer for 20 min, vortexing regularly. Immunoprecipitated proteins were separated on SDS–PAGE acrylamide gels, and western blots were performed using anti-GFP (Santa Cruz Biotechnology sc-9996), anti-SYT1 (Pérez-Sancho *et al.*, 2015), anti-mouse IgG-peroxidase (Sigma A9044), and anti-rabbit IgG-peroxidase (Sigma A0545).

### Molecular cloning and generation of transgenic plants

The *CLB1* and *SYT5* constructs used in this study were generated via PCR amplification using the reverse transcription–PCR (RT–PCR) product or genomic DNA as a template and gene-specific primers (Supplementarty Table S2 at *JXB* online), followed by cloning PCR products into pENTR/TOPO (Invitrogen, Carlsbad, CA, USA) or pDONR221. To generate the *pUB10::CLB1-GFP* construct, the CLB1 fragment was subcloned into the pB7m24GW,3 vector that contains a 615 bp UBQ10 promoter. To generate the p35S:SYT1-N-GFP, p35S:SYT1-C-GFP, p35S:SYT5-N-GFP, and p35S:SYT5-C-GFP constructs used in bimolecular fluorescence complementation (BiFC), combinations of pEN-L4-pro35S-R1 (Karimi *et al.*, 2007), pEN-L4-proSYT5-R1, pEN-R2-N-GFP-L3 (Boruc *et al.*, 2010), and pEN-R2-C-GFP-L3 (Boruc *et al.*, 2010) were recombined with pEN-L1-SYT1genomic-L2 (Pérez-Sancho *et al.*, 2015) and pEN-L1-SYT5genomic-L2 into pK7m34GW,0 (Karimi *et al.*, 2005). To generate the pCLB1::CLB1-GFP construct, the CLB1 pENTR clone was recombined with the destination binary vector pGWB4. All resulting expression vectors were transformed in Arabidopsis via floral dip (Clough and Bent, 1998). The selection of transgenic lines was made on half-strength MS medium containing 25 μg ml^–1^ hygromycin (pGWB4) or 15 μg ml^–1^ glufosinate-ammonium (Sigma-Aldrich) (pB7m24GW,3).

### Chemical applications

Chemicals were exogenously applied by incubating 5-day-old seedlings in liquid 1/10th strength MS medium and supplementing them with 500 µM LaCl_3_ (Sigma-Aldrich), 500 µM GdCl_3_ (Sigma-Aldrich), 5 mM EGTA (Sigma-Aldrich), or 25 μM oryzalin (Sigma-Aldrich) for 2 h or 16 h, or with 250 µM bis-(*o*-aminophenoxy) ethane-*N*,*N*,*N'*,*N'*-tetra-acetic acid (BAPTA) (Sigma-Aldrich) or 1 µM latrunculin B (Abcam) for 2 h or 16 h. The duration of the treatments was based on the general toxicity caused by the different chemical compounds in plants. To visualize Hechtian strands, 5-day-old cotyledon epidermal cells expressing the different markers were plasmolyzed for 4 h using 0.4 M mannitol. The images are an overlay of propidium iodide-stained cell walls with the localization of the GFP fusion proteins in green.

### Image acquisition and quantitative analyses

Living cell images were obtained using a Nikon C1 confocal laser scanning microscope, a Perkin-Elmer spinning disk confocal microscope, and an Olympus FV1000 multiphoton confocal laser scanning microscope. The Nikon C1 confocal laser scanning microscope was equipped with 488 nm and 515/30 nm emission filters and Nikon Plan Apochromat oil immersion objectives (×40, 1.0 NA and ×60, 1.4 NA, respectively). The Perkin-Elmer spinning disk confocal microscope was equipped with 488 nm and 561 nm lasers. The Olympus FV1000 was equipped with 405, 473, and 559 nm lasers and a ×60 oil Planon (×60, 1.4 NA). Images were captured using Nikon-EZ C1, Olympus FV1000, and Volocity software, respectively. To quantify the number of ‘beads’ configuration, 5-day-old Arabidopsis seedlings harboring the SYT1–GFP or SYT5–GFP marker were incubated for 16 h in liquid 1/10th strength MS medium (Mock) or liquid 1/10th strength MS medium supplemented with the different chemicals. For each treatment, the number of ‘beads’ labeled by SYT1–GFP or SYT5–GFP in the cortex of cotyledon epidermal cells was scored in at least 50 (15 μm×15 μm) regions of interest (ROIs) using the cell counter tool of Fiji (ImageJ) (National Institutes of Health, http://imagej.nih.gov/ij/) (Schindelin *et al.*, 2012). To compare the fluorescent intensity of the ratiometric CITRINE-1×PH^FAPP^ between control and treated samples, confocal laser scanning images of 5-day-old epidermal cotyledon cells were acquired from at least 10 individual seedlings. For each data point, the fluorescence intensity data were scored from at least 100 (15 μm×15 μm) ROIs using Fiji’s integrated density measurement tool (Schindelin *et al.*, 2012). In this analysis, stomatal lineage cells were excluded from the quantification. To compare the fluorescent intensity of the ratiometric GCaMP3 sensor, images of 5-day-old seedlings were acquired using a Nikon SMZ18 stereo microscope equipped with a 480/40 nm excitation filter, a Nikon P2-SHR Plan Apo ×0.5 objective, and a Nikon DS-Ri2 camera. The images were captured using NIS-Elements BR software version 4.60. For each data point, the fluorescence intensity data were scored from at least 50 seedlings. In the ratiometric analyses, the fluorescent data were normalized using the equation: ∆*F*/*F*=(*F*–*F*_0_)/*F*_0_, where *F*_0_ is the mean intensity of background fluorescence. The data were subject to one-way ANOVA to identify statistically significant differences among treatments. All statistical analyses were performed using the GraphPad Prism 5.0b software.

### Accession numbers

The Arabidopsis Genome Initiative locus identifiers for genes used in this article are SYT1 (*At2g20990*), SYT5 (*At1g05500*), and CLB1 (*At3g61050)*.

## Results

### SYT5 and CLB1 are EPCS-localized proteins that interact with SYT1 *in vitro*

**Fig. 1.**
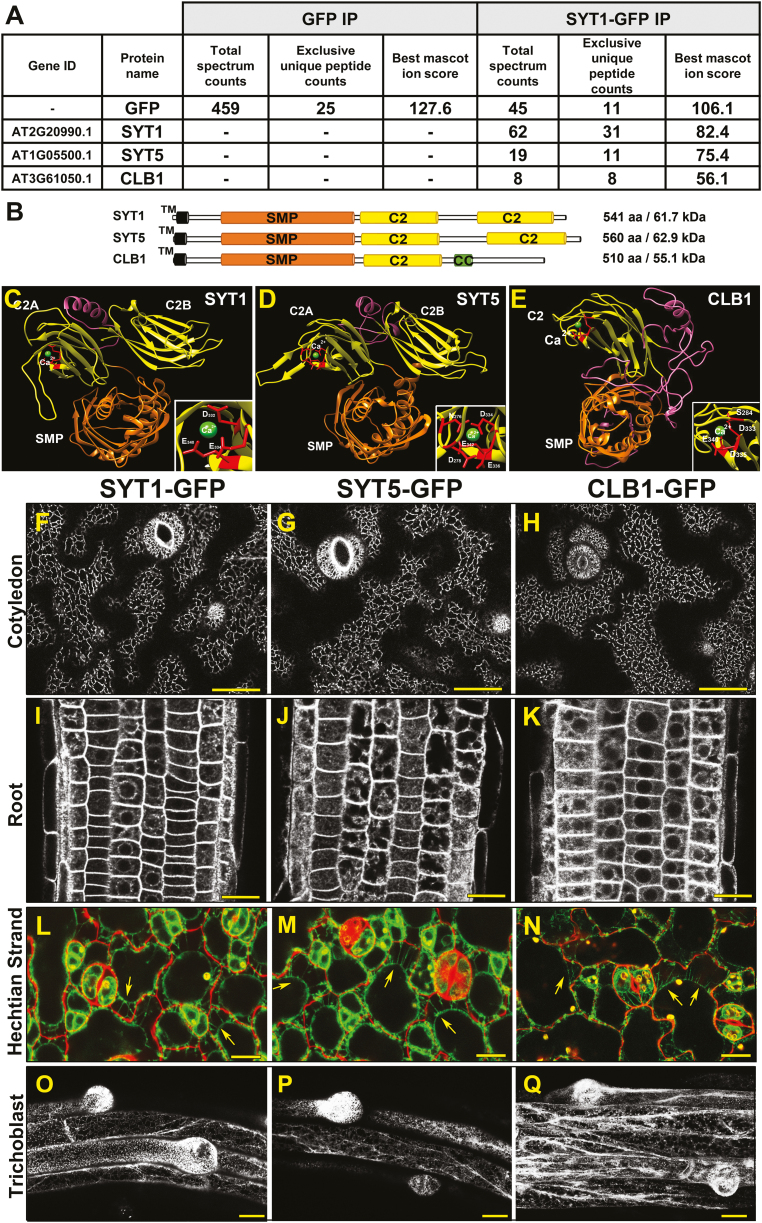
The Ca^2+^-dependent phospholipid-binding proteins SYT5 and CLB1 interact with SYT1. (A) Peptide counts detected upon GFP immunoprecipitation followed by LC-MS/MS analysis using Arabidopsis plants expressing GFP (control) and SYT1–GFP. Numbers indicate the total spectrum counts corresponding to the indicated proteins, and the exclusive unique peptides represented within them. The best Mascot ion score among these peptides is indicated. The number of peptides corresponding to GFP is shown for reference. This result is representative of three independent experiments (for details on the replicates, see Supplementary Table S1). (B) Schematic representation of the functional domains of SYT1, SYT5, and CLB1. TM, transmembrane domain; SMP, synaptotagmin-like mitochondrial-lipid binding domain; C2, phospholipid-binding domains; CC, coiled-coil domain. (C–E) 3D structures and Ca^2+^-binding sites in the predicted cytosolic regions of SYT1 (C), SYT5 (D), and CLB1 (E) identified using Phyre2 and 3DLigand site. Important amino acid residues for Ca^2+^ binding are indicated in red. (F–Q) Subcellular localization of the SYT1–GFP, SYT5–GFP, and CLB1–GFP markers in epidermal cells of 5-day-old cotyledons (F–H), root meristematic cells of 5-day-old seedlings (I–K), Hechtian strands in plasmolyzed cells (L and M arrows), and emerging root hairs in 7-day-old seedlings (O–Q). Scale bars (F–K)=25 μm; (L–M)=20 μm; and (O–Q)=25 μm.

SYT1 is a protein tether implicated in the establishment, organization, and function of plant EPCSs (Pérez-Sancho *et al.*, 2016a; Tilsner *et al.*, 2016; Bayer *et al.*, 2017; Wang *et al.*, 2017). Because the SYT1 orthologs in mammals [extended synaptotagmins (E-Syts)] and yeast [tricalbins (Tcbs)] establish tethering complexes *in vivo* (Creutz *et al.*, 2004; Giordano *et al.*, 2013), we searched for additional proteins physically associated with Arabidopsis SYT1. For this purpose, we used a SYT1–GFP line in the *syt1-2* background (Lee *et al.*, 2019) and performed IP assays using agarose beads coupled to an anti-GFP nano-body (GFP-Trap beads). The IP results from three independent biological replicates provided a large number of proteins physically associated with SYT1 that we identified using LC-MS/MS. We filtered the results using the following criteria: (i) presence in all three biological replicates; (ii) detection of two or more exclusive unique peptides; and (iii) absence in the negative IP control (IP using a transgenic line expressing free GFP). After applying these filters, we identified two putative SYT1 interactors: Arabidopsis SYT5 (*At1g05500*; Ishikawa *et al.*, 2020) and CLB1/SYT7 (*At3g61050*; de Silva *et al.*, 2011; Ishikawa *et al.*, 2020) (Fig. 1A; Supplementary Table S1).

Bioinformatics analyses using Pfam (El-Gebali *et al.*, 2019) and TMHMM2 (Krogh *et al.*, 2001) databases show that SYT1, SYT5, and CLB1 share common domain architectures comprising a putative single N-terminal TM domain, an ~40 amino acid linker, a cytoplasm-exposed synaptotagmin-like mitochondrial lipid-binding protein (SMP) domain, and one (CLB1) or two (SYT1 and SYT5) phospholipid-binding C2 domains harboring lysine/arginine-rich (K/R-rich) polybasic patches (Fig. 1B; Supplementary Fig. S1). 3D modeling using Phyre2 (Kelley *et al.*, 2015) and 3DLigandSite (Wass *et al.*, 2010) shows that the predicted cytosolic regions of these proteins (SYT1_30–541_, SYT5_23–560_, and CLB1_22–510_) can be modeled with >90% confidence using the crystal structure of the mammalian E-Syt2 as a template (Fig. 1C–E). The modeling also shows that SYT1, SYT5, and CLB1 contain a single Ca^2+^-binding site, whose position could be determined with confidence levels above the 99% threshold (Fig. 1C–E insets).

To assess the subcellular localization of the SYT5 and CLB1 proteins, we generated fluorescent SYT5–GFP and CLB1–GFP marker lines driven by their respective endogenous promoters. We used confocal microscopy and compared the SYT5–GFP and CLB1–GFP subcellular localization with that of the SYT1–GFP marker (Lee *et al.*, 2019). Figure 1F–Q shows that the SYT5–GFP and CLB1–GFP localization strongly resembles that of the SYT1–GFP marker in all tissues analyzed. These localizations include a ‘beads and strings’ arrangement in cotyledon epidermal cells (Fig. 1F–H), perinuclear labeling consistent with the ER in root meristematic cells (Fig. 1I–K), associations with the cell wall through Hechtian strands (Fig. 1L–N), and strong signal accumulation at root hair initiation sites (Fig. 1O–Q). The latter localization is consistent with a putative function for the SYT1/SYT5/CLB1 complex in root hair polarity maintenance, as indicated by the root hair phenotypes in the presence of NaCl of the *syt1/syt5/clb1* triple mutant (Supplementary Fig. S2). Although confocal microscopy alone is not sufficient to establish unequivocally whether the observed subcellular localizations represent EPCSs, the protein interaction data, the shared structural and functional features, and the common localization patterns strongly suggest that, like SYT1, SYT5 and CLB1 are enriched at EPCSs.

### SYT1 and SYT5 establish homotypic and heterotypic interactions *in vivo* at EPCSs

**Fig. 2.**
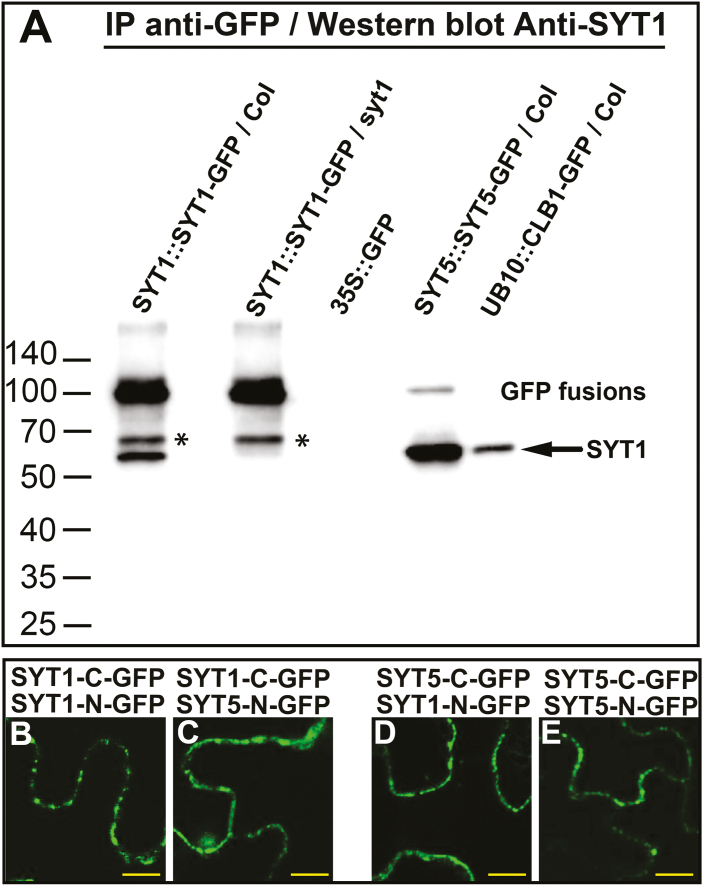
SYT1 and SYT5 form homo- and heterodimeric complexes. (A) Arabidopsis transgenic plants expressing SYT1–GFP, SYT5–GFP, and CLB1–GFP were used for immunoprecipitation using GFP-Trap beads. Plants expressing free GFP were used as control. The immunoprecipitated proteins were separated by SDS–PAGE, and western blots were analyzed using anti-SYT1. Molecular weight (kDa) marker bands are indicated for reference. The arrow indicates the expected molecular weight for SYT1. *indicate a SYT1–GFP-derived fragment recognized by the SYT1 antibody. (B–E) BiFC analyses of SYT1/SYT5 interactions. *Nicotiana benthamiana* leaves were co-transformed transiently with the following SYT1 and SYT5 combinations: SYT1-C–GFP and SYT1-N–GFP (B), SYT1-C–GFP and SYT5-N–GFP, (C), SYT5-C–GFP and SYT1-N–GFP, (D), and SYT5-C–GFP and SYT5-N–GFP (E), and imaged after 4 d. Scale bars=10 μm.

To validate the interactions between SYT1, SYT5, and CLB1, we used a targeted co-IP assay using a previously reported anti-SYT1 polyclonal antibody (Pérez-Sancho *et al.*, 2015). For this experiment, we used the SYT1–GFP line in the *syt1-2* background (Lee *et al.*, 2019), a SYT1–GFP line in the Col background (Pérez-Sancho *et al.*, 2015), and we generated a transgenic line expressing SYT5–GFP under its native promoter (*SYT5::SYT5-GFP*) and a transgenic line expressing CLB1–GFP under a constitutive ubiquitin 10 promoter (*pUB10::CLB1-GFP*). Figure 2A and Supplementary Fig. S3 show that the affinity-purified SYT1–GFP was able to pull-down the native SYT1 (lane 1, 61.7 kDa band) from protein extracts *in vitro*, and that this interaction was not present when the SYT1–GFP line in the *syt1-2* background was used (lane 2). SYT5–GFP and CLB1–GFP were also able to pull-down the native SYT1 from protein extracts (lanes 4 and 5, 61.7 kDa band). Next, we assessed the putative interaction of these proteins *in vivo* using BiFC assays. Figure 2B–E shows that transient co-expression of different SYT1 and SYT5 BiFC constructs in *Nicotiana benthamiana* leaves render BiFC signals consistent with SYT1 and SYT5 interacting and forming homo- and heterodimers at PM subdomains. Despite multiple attempts, we failed to observe a BiFC signal between SYT1 and CLB1, and focused our subsequent analyses on SYT1 and SYT5. During the revision of this study, Ishikawa *et al.* (2020) reported the interaction between SYT1 and CLB1 *in vivo* using BiFC.

### The putative SYT1/SYT5 EPCS complex is largely insensitive to extracellular Ca^2+^ depletion but relocalizes in response to internalization of REEs

**Fig. 3.**
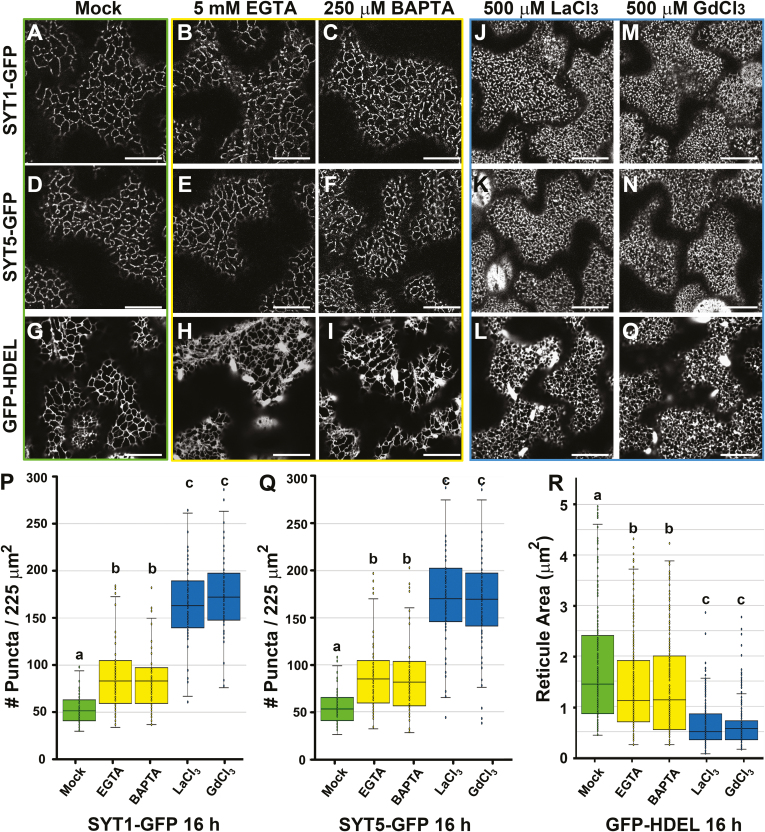
Extracellular Ca^2+^ depletion and REE treatments increase the EPCS number and reduce cortical ER reticulation. Five-day-old SYT1–GFP, SYT5–GFP, and HDEL–GFP seedlings were treated in liquid 1/10th strength MS medium supplemented with Mock (A–C), EGTA (5 mM) (D–F), BAPTA (250 µM) (G–I), LaCl_3_ (500 µM) (J–L), or GdCl_3_ (500 µM) (M–O) for 16 h prior to imaging and quantification. (P and Q) Quantification of SYT1–GFP and SYT5–GFP puncta at the cell cortex upon chemical treatments. (R) Quantification of HDEL–GFP reticulation. In (P–R), the number of puncta or closed reticules was scored using 50–60 arbitrary 225 µm^2^ ROIs from at least 15 cells from five independent seedlings. In the box and whiskers plots, the center line represents the median number of puncta or closed reticules per 225 µm^2^, the top and bottom edges are the 25th and 75th percentiles of the distribution, and the ends of the whiskers are set at 1.5 times the interquartile range (IQR). All values outside the IQR are shown as outliers (dots). Letters indicate statistically significant differences using Tukey multiple pairwise comparisons *P*<0.05. Scale bars=20 μm.

The SYTs orthologs in yeast and mammals are Ca^2+^-responsive proteins that sense changes in [Ca^2+^]_cyt_ and regulate the non-vesicular transfer of signaling molecules between the cortical ER and the PM (Creutz *et al.*, 2004; Giordano *et al.*, 2013; Helle *et al.*, 2013; Prinz, 2014). Because SYT1, SYT5, and CLB1 contain a putatively conserved Ca^2+^-binding site in their 3D structure, we asked whether Ca^2+^ signals could influence the localization and dynamics of the SYT1/SYT5 tethering complex. To address this question, we first analyzed the effect of extracellular Ca^2+^ depletion on SYT1–GFP and SYT5–GFP localization using the extracellular Ca^2+^-chelating agents EGTA and BAPTA (Brault *et al.*, 2004; Nakagawa *et al.*, 2007) at different time points. In 2 h treatments, the depletion of free apoplastic Ca^2+^ induced by either EGTA or BAPTA does not have a significant effect on the number of SYT1–GFP- and SYT5–GFP-labeled ‘beads’ at the cell cortex (Supplementary Fig. S4). In 16 h treatments, EGTA and BAPTA induced an ~1.5- to 1.8-fold increase in the number of SYT1–GFP- and SYT5–GFP-labeled ‘beads’ and a reduction in the average reticule size of the cortical ER network (Fig. 3A–F, R). We also tested the effect of La^3+^ and Gd^3+^ REEs on SYT1–GFP and SYT5–GFP localization at different time points. Supplementary Fig. S5 shows that the Ca^2+^ channel-blocking activity of REEs in 30 min treatments did not induce changes in SYT1–GFP and SYT5–GFP localization. Remarkably, 2 h treatments induced a variable 2- to 4-fold increase in the number of SYT1–GFP- and SYT5–GFP-labeled ‘beads’ in individual epidermal cells (Supplementary Fig. S4), and 16 h treatments induced a generalized 4- to 5-fold increase in the number of SYT1–GFP- and SYT5–GFP-labeled ‘beads’ in all epidermal cells (Fig. 3J–Q). The treatment was associated with a significant reduction of the average reticule size of the cortical ER network (Fig. 3R), and with an increase in the number of ‘beads’ labeled by the artificial EPCS marker MAPPER–GFP (Lee *et al.*, 2019) (Supplementary Fig. S6). Remarkably, a 16 h La^3+^ treatment did not induce major changes in the localization of a truncated SYT1–GFP marker harboring the C2 phospholipid-binding domains (C2AB–GFP; Pérez-Sancho *et al.*, 2015) that is still homogeneously distributed at the PM (Supplementary Fig. S7).

**Fig. 4.**
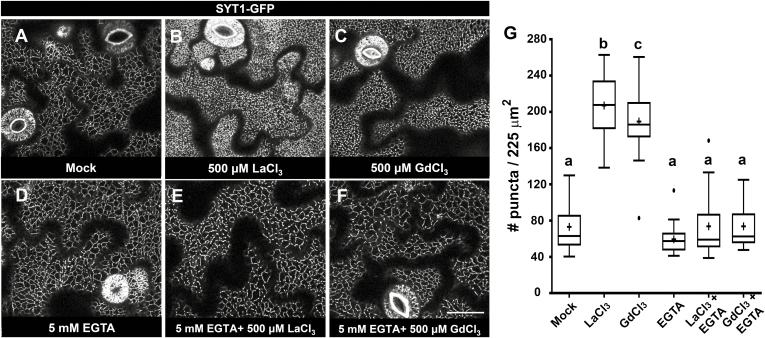
The addition of EGTA to the growth medium reduces the SYT1–GFP and SYT5–GFP localization changes associated with REE internalization. Five-day-old SYT1–GFP seedlings were treated in liquid 1/10th strength MS medium supplemented with Mock (A), LaCl_3_ (500 µM/16 h), (B) GdCl_3_ (500 µM/16 h) (C), or the same medium supplemented with 5 mM EGTA (D–F) before imaging. (G) Quantification of the SYT1–GFP cortical signal. For each treatment, the number of puncta was scored using 50–60 arbitrary 225 µm^2^ ROIs from at least 15 cells from five independent seedlings. In the box and whiskers plots, the center line represents the median number of puncta per 225 µm^2^, the top and bottom edges are the 25th and 75th percentiles of the distribution, and the ends of the whiskers are set at 1.5 times the interquartile range (IQR). When present, the minimum/maximum values outside the IQR are shown as outliers (dots). Letters indicate statistically significant differences using Tukey multiple pairwise comparisons *P*<0.05. Scale bar=20 μm.

The dynamics of SYT1–GFP and SYT5–GFP relocalization are consistent with EPCS remodeling being triggered by the slow internalization of REEs to the cytosol (L. Wang et al., 2014, 2016, 2019). In this scenario, the addition of EGTA, a polydentate chelator that forms stable complexes with both Ca^2+^ and REEs (Tei *et al.*, 2010), should maintain REEs in the extracellular space and prevent the REE-induced SYT1–GFP and SYT5–GFP relocalization. Figure 4A–E shows that the supplementation of the REE treatments with 5 mM EGTA was sufficient to abolish the REE-induced SYT1–GFP relocalization in 16 h treatments, and reduce the long-term toxicity of the REEs in seedlings treated for 14 d (Supplementary Fig. S8). These results suggest that REE internalization, and not the blockage of extracellular Ca^2+^ entry induced by either REEs or Ca^2+^ chelators, underlies EPCS remodeling.

### Internalized REEs can act as Ca^2+^ signaling surrogates in the cytosol

**Fig. 5.**
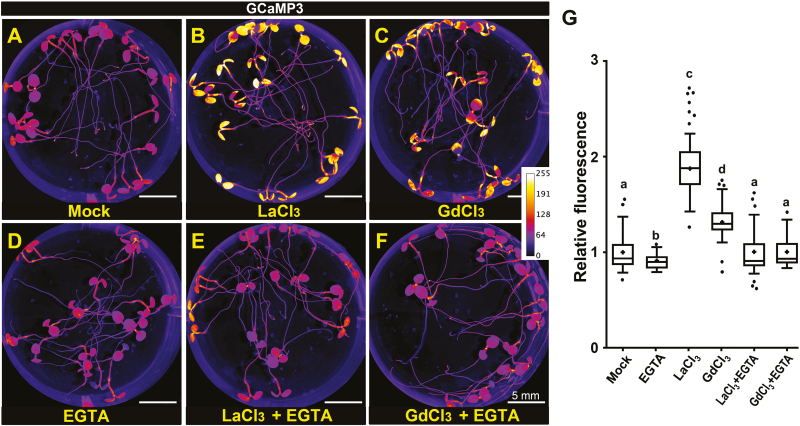
REEs induce the activation of the cytosolic GCaMP3 Ca^2+^ sensor. Fluorescence images of seedlings expressing the GCaMP3 Ca^2+^ sensor. Five-day-old seedlings were treated in liquid 1/10th strength MS medium supplemented with Mock, 16 h (A), LaCl_3_ (500 µM/16 h) (B), GdCl_3_ (500 µM/16 h) (E), or the same medium supplemented with 5 mM EGTA (D–F). The activity of the Ca^2+^ sensor is shown as color-coded pixel intensity following the LUT scale shown in (F). (G) Quantification of the GCaMP3 signal relative to mock conditions. The center line represents the median fluorescence intensity fold increase relative to mock, the cross represents the mean fluorescent intensity, the top and bottom edges are the 25th and 75th percentiles of the distribution, and the ends of the whiskers are set at 1.5 times the interquartile range (IQR). All values outside the IQR are shown as outliers. The intensity of the signal was measured for at least 50 seedlings per treatment. Letters indicate statistically significant differences using Tukey multiple pairwise comparisons *P*<0.05. Scale bar=5 mm.

Biochemical studies have shown that REEs act as allosteric regulators of multiple Ca^2+^-binding proteins *in vitro* (Mills and Johnson, 1985; Bertini *et al.*, 2003; Ye *et al.*, 2005; L. Wang *et al.*, 2016), so we asked whether internalized REEs could replace Ca^2+^ and mimic its effect *in vivo*. To answer this question, we analyzed the effect of REEs on the cytosolic activity of the calmodulin-based ratiometric Ca^2+^ sensor GCaMP3 (Tian *et al.*, 2009). Figure 5A–G shows that 16 h REE treatments that promote their internalization also induce a 2- to 3-fold increase in the GCaMP3 fluorescent signal in a process that is abolished by 5 mM EGTA supplementation. These results are consistent with internalized REEs acting as Ca^2+^ surrogates and binding proteins containing Ca^2+^/calmodulin-like binding domains.

### The La^3+^-induced relocalization is cytoskeleton dependent and it is associated with PI4P accumulation at the PM

**Fig. 6.**
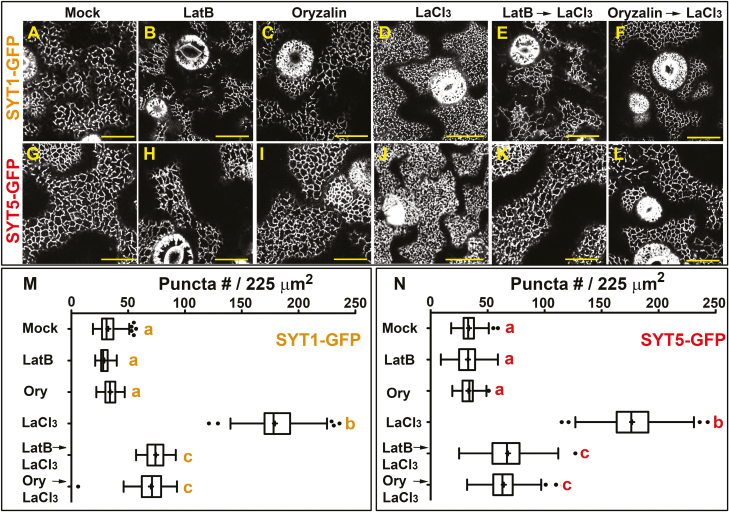
REEs induce cytoskeleton-dependent changes in EPCS configuration. Confocal images of the cell cortex in cotyledon epidermal cells expressing the SYT1–GFP (A–F) and SYT5–GFP (G–L) markers. Five-day-old transgenic seedlings grown in 1/10th MS were transferred to liquid 1/10th MS for 16 h (A and G), or the same medium supplemented with LatB (1 μM, 2 h) (B and H), oryzalin (25 μM, 16 h) (C and I), LaCl_3_ (500 μM, 16 h) (D and J), or sequentially treated with LatB (1 μM, 2 h) followed by LaCl_3_ (500 μM, 16 h) (E and K) or oryzalin (25 μM, 16 h) followed by LaCl_3_ (500 μM, 16 h) (F–L) before imaging. (M and N) Quantification of SYT1–GFP and SYT5–GFP puncta at the cell cortex upon chemical treatments For each treatment, the number of SYT1–GFP and SYT5–GFP puncta was scored from 50–60 arbitrary 225 µm^2^ ROIs from at least 15 cells from five different seedlings. In the box and whiskers plots, the center line represents the median number of puncta per 225 μm^2^, the top and bottom edges are the 25th and 75th percentiles of the distribution, and the ends of the whiskers are set at 1.5 times the interquartile range (IQR). the minimum maximum values outside the IQR are shown as outliers (dots). Letters indicate statistically significant differences using Tukey multiple pairwise comparisons *P*<0.05 Scale bars=20 μm.

**Fig. 7.**
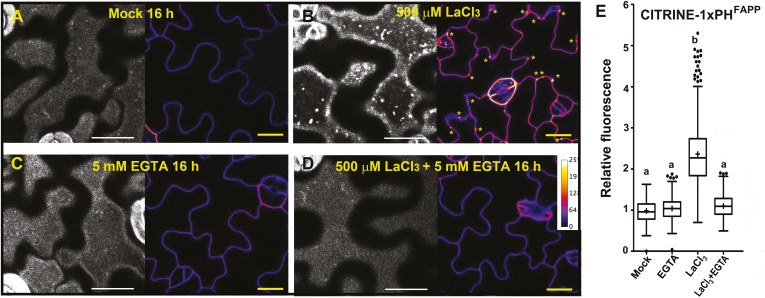
La^3+^ induces the accumulation of PI4P at the PM and the formation of PI4P-containing vesicles. (A–D) Confocal images of the cell cortex and equatorial planes of cotyledon epidermal cells expressing the CITRINE-1×PH^FAPP^ marker. Five-day-old seedlings were treated in liquid 1/10th strength MS medium supplemented with Mock, 16 h (A), LaCl_3_ (500 µM/16 h) (B), EGTA (5 mM/16 h) (C), or LaCl_3_ (500 µM)+EGTA (5 mM)/16 h (D). The presence of PI4P-labeled vesicles is shown as bright dots at the PM (yellow asterisks). PI4P accumulation is shown as color-coded pixel intensity following the LUT scale shown in (D). (E) Quantification of the CITRINE-tagged 1×PH^FAPP^ signal relative to mock conditions. In the box plots, the center line represents the median fluorescence intensity fold increase relative to mock, the cross represents the mean fluorescent intensity, the top and bottom edges are the 25th and 75th percentiles of the distribution, and the ends of the whiskers are set at 1.5 times the interquartile range (IQR). All values outside the IQR are shown as outliers. At least 100 regions of interest (ROIs) were measured for each treatment. The letters indicate statistically significant differences using Tukey multiple pairwise comparisons *P*<0.05. Scale bars=20 μm.

**Fig. 8.**
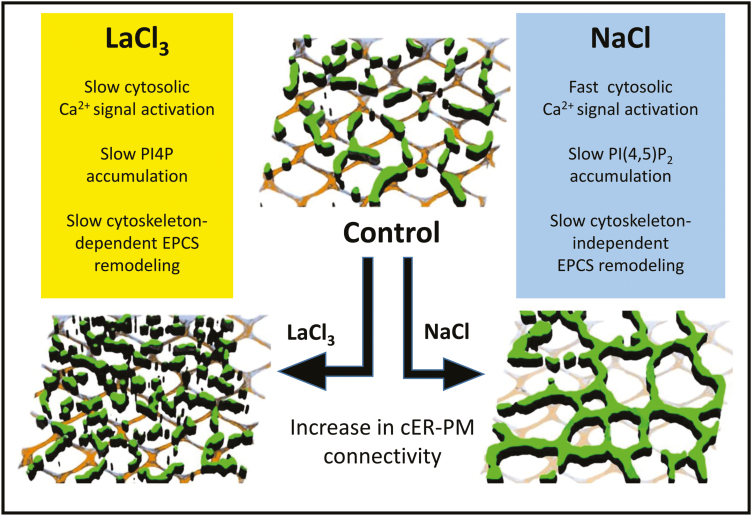
Comparison of the mechanisms underlying LaCl_3_- and NaCl-induced EPCS remodeling. In this representation, the green signal represents the localization of the SYT1–GFP or SYT5–GFP markers at the cell cortex, and the crisscross pattern represents the cortical cytoskeleton.

In the final experiment, we used 500 μM La^3+^ and 100 mM NaCl treatments for 16 h to explore whether the REE-induced EPCS reorganization is mechanistically similar to that previously reported for NaCl stress (Lee *et al.*, 2019). First, we tested whether the cortical cytoskeleton plays a role in the La^3+^-induced EPCS remodeling. Our results show a differential behavior between the treatments as, compared with NaCl stress (Lee *et al.*, 2019), La^3+^ does not cause visible disruption of the cortical cytoskeleton network (Supplementary Fig. S9), and its effect on EPCS organization is partially abolished by pre-treatments with the microtubule-depolymerizing drug oryzalin or the actin polymerization inhibitor latrunculin B (Fig. 6A–N). Next, we tested whether the La^3+^-induced EPCS remodeling was associated with the accumulation of phosphoinositides at the PM using the ratiometric sensors citrine 1×PH^FAPP1^ (for PI4P) and 2×PH^PLC^ [for PI(4,5)P_2_] (Simon *et al.*, 2016). Our results show that 16 h La^3+^ treatments do not induce accumulations of the PI(4,5)P_2_ sensor (Supplementary Fig. S10), but did induce an ~2-fold increase of the PI4P fluorescent signal at the PM (Fig. 7A, B, E). Remarkably, the 16 h La^3+^ treatment also induced the formation of PI4P-labeled vesicle-like structures closely associated with the PM (Fig. 7B asterisks). Consistent with previous findings, the addition of 5 mM EGTA to the extracellular medium was sufficient to inhibit the La^3+^-induced PI4P accumulation and the formation of PI4P vesicles (Fig. 7C, D). The results support a model where stress-induced accumulations of specific phosphoinositides is associated with either cytoskeleton-dependent or cytoskeleton-independent rearrangements of EPCS-localized protein complexes (Fig. 8).

## Discussion

### The plant EPCS responses to [Ca^2+^]_cyt_ are unique among eukaryotes

EPCSs are ubiquitous structures in eukaryotes, and they adopt distinct shapes and architectures in response to environmental and developmental cues. In mammals and yeast, EPCSs have a well-known role in the control of Ca^2+^ dynamics but, in plants, the presence of a cell wall that maintains a high extracellular Ca^2+^ concentration, and the presence of a complex suite of Ca^2+^ channels, transporters, and signaling components (Wheeler and Brownlee, 2008; De Vriese *et al.*, 2018) has limited our understanding of the role of Ca^2+^ in cortical ER–PM communication. Mammalian E-Syts and plant SYT1/SYT5 EPCS complexes share a common basic organization as both establish homotypic and heterotypic protein-tethering complexes with their N-terminal domains anchored to the ER, and their C-terminal C2 domains establishing Ca^2+^-dependent interactions with the PM (Giordano *et al.*, 2013; Pérez-Sancho *et al.*, 2015; this study). Intriguingly, the mammalian E-Syts aggregate and concentrate at membrane junctions following a rise in [Ca^2+^]_cyt_ (Giordano *et al.*, 2013), and this behavior is replicated by SYT1 in response to La^3+^ treatments. To explain this observation, Ishikawa *et al.* (2018) proposed that SYT1 responds to a decrease, instead of an increase, in [Ca^2+^]_cyt_ due to the activity of La^3+^ as a Ca^2+^ channel blocker at the PM. Our results reconcile this seemingly contrasting behavior in plants and mammals by showing that, in long-term treatments, internalized REEs are capable of triggering intracellular Ca^2+^ signals, effectively offsetting their effect as PM Ca^2+^ channel blockers. Given that REEs can act as allosteric regulators of the activity of calmodulins (Mills and Johnson, 1985; L. Wang *et al.*, 2016), and C2-containing proteins *in vitro* (Essen *et al.*, 1997), we propose that internalized REEs could facilitate slow changes in ER–PM communication either by activating calmodulin signaling or through direct binding to the SYT1, SYT5, and/or CLB1 Ca^2+^-binding domains *in vivo*.

The results discussed above highlight a clear difference in the temporal regulation of the Ca^2+^-mediated responses between mammalian E-Syts and plant SYTs. In non-excitable mammalian cells, EPCSs control intracellular Ca^2+^ levels using store-operated Ca^2+^ entry (SOCE), a fast process that couples the Ca^2+^ influx from the extracellular space to the cytosolic Ca^2+^ release from the ER within seconds (Orci *et al.*, 2009). These mammalian cells can also sense high [Ca^2+^]_cyt_ and trigger the recruitment of E-Syt1 tethers to SOCE-independent EPCSs within minutes (Wu *et al.*, 2006). In contrast, the depletion of extracellular Ca^2+^ by chelating agents in Arabidopsis has limited effect on SYT1–GFP and SYT5–GFP localization, and EPCS remodeling in response to REEs and NaCl takes place within hours (this study; Lee *et al.*, 2019). Based on these observations, we hypothesize that the plant SYT1/SYT5 complexes are involved neither in the fast coupling of the extracellular and ER–lumen Ca^2+^ stores, nor in the fast response to [Ca^2+^]_cyt_ changes induced by stress. Instead, we propose that the observed EPCS remodeling in response to REEs is a consequence of the sensing and transduction of stress signals that promote long-term cellular adaptive responses, such as the slow changes in the PM lipid composition discussed in the next section.

### Stress-specific regulatory mechanisms controlling EPCS organization in Arabidopsis

The cortical ER is a complex arrangement of tubules and small cisternae distributed towards the PM (Stefano *et al.*, 2014; Griffing *et al.*, 2017). EPCSs are important substructures within the cortical ER that can be defined as 200–300 nm long and 30 nm wide cortical ER nanodomains, which anchor to the PM using specialized tethering complexes (McFarlane *et al.*, 2017). In a differentiated plant cell, EPCSs can be localized in immobile ER tubules (Ishikawa *et al.*, 2018), and are associated with the cortical cytoskeleton (Peña and Heinlein, 2013; P. Wang et al., 2014, 2016; Lee *et al.*, 2019). Currently, two functions of the cortical cytoskeleton array in EPCS establishment have been proposed. On the one hand, the actin and microtubule networks physically interact with VAP27/NET3C tethering complexes, fixing them on specific positions within the cell cortex (P. Wang et al., 2014, 2016). This interaction might be required for cargo exchange during endocytic and exocytic trafficking (Peña and Heinlein, 2013; P. Wang *et al.*, 2014). On the other hand, the cortical cytoskeleton is required for the delivery of SYT1 tethers to EPCSs, and could also generate spatial incompatibility for EPCS establishment in regions where ‘thick’ cortical microtubules (25 nm in diameter) are closely associated with the PM (Pérez-Sancho *et al.*, 2015; McFarlane *et al.*, 2017; Lee *et al.*, 2019).

Given that SYT1/SYT5 complexes require a functional cortical cytoskeleton for proper reorganization in response to REEs, we hypothesize that their activity could be coordinated with that of the VAP27/NET3C EPCS complexes. In response to REE stress, the VAP27/NET3C and SYT1/SYT5 complexes could integrate cytoskeleton dynamics, cortical ER stability, and EPCS positioning, effectively controlling cortical ER–PM communication. In this context, the REE-induced PI4P accumulation at the PM could influence the electrostatic surface of the PM (Simon *et al.*, 2016), and regulate the docking affinity of the EPCS tethering complexes. Whether these changes in lipid composition could also activate endocytic and/or autophagic processes at EPCSs as proposed in Wang and Hussey (2019) has not been established and it is an area of active research in our laboratory. Remarkably, in plants subject to stress conditions that induce cytoskeleton disassembly (e.g. NaCl), an alternative SYT1-dependent mechanism promotes cytoskeleton-independent EPCS remodeling (Lee *et al.*, 2019). In these conditions, the NaCl-induced accumulation of PI(4,5)P_2_ at the PM would have a minor influence on the PM electrostatic field, as PI4P still acts as the main contributor in this process (Simon *et al.*, 2016), but could fine-tune EPCS-associated signaling pathways acting as a substrate of PM-localized phospholipases (e.g. PI-PLCs) (Singh *et al.*, 2015). Together, these mechanisms illustrate the specificity and plasticity that govern EPCS rearrangements as an adaptive response to environmental stresses (Fig. 8).

## Supplementary data

Supplementary data are available at *JXB* online.

Table S1. Unique peptide counts in three independent immunoprecipitation experiments.

Table S2. Primers used in this study.

Fig. S1. Multiple sequence alignment of the SYT1/SYT5/CLB1 C2 domains.

Fig. S2. Root hair polarization defects in the triple *syt1/syt5/clb1* mutant.

Fig. S3. Expression control for immunoprecipitation experiments.

Fig. S4. Effect of 2 h extracellular Ca^2+^ depletion and REE treatments on EPCS number.

Fig. S5. Effect of short-term REE treatments on EPCS number.

Fig. S6. Effect of REE treatments on MAPPER–GFP localization.

Fig. S7. Effect of La^3+^ treatments on the localization of the PM marker C2AB–GFP.

Fig. S8. Effect EGTA supplementation on REE-induced seedling growth defects.

Fig. S9. Effect of NaCl and LaCl_3_ treatments on cortical cytoskeleton organization.

Fig. S10. Effect of La^3+^ treatments on the accumulation of PI(4,5)P_2_ at the PM.

eraa138_suppl_supplementary_table_S1-S2_figures_S1-S10
