# Supplementary material for: Rare earth elements induce cytoskeleton-dependent and PI4P-associated rearrangement of SYT1/SYT5 endoplasmic reticulum–plasma membrane contact site complexes in Arabidopsis
Source: J Exp Bot. 2020 Mar 17;71(14):3986–98. doi: 10.1093/jxb/eraa138 (PMC7337092; doi:10.1093/jxb/eraa138)
Supplement: eraa138_suppl_supplementary_table_S1-S2_figures_S1-S10 [file eraa138_suppl_supplementary_table_s1-s2_figures_s1-s10.pdf]

Supplemental Data

		Replicate 1		Replicate 2		Replicate 3	
Gene ID	Protein name	GFP IP	SYT1-GFP IP	GFP IP	SYT1-GFP IP	GFP IP	SYT1-GFP IP
-	**GFP**	25	11	24	18	29	24
**AT2G20990.1**	**SYT1**	-	31	-	56	-	68
**AT1G05500.1**	**SYT5**	-	11	-	23	-	39
**AT3G61050.1**	**CLB1**	-	8	-	16	-	12
		Exclusive unique peptide counts					

Table S1: SYT5 and CLB1 are reproducible interactors of SYT1. The table shows exclusive unique peptide counts corresponding to the indicated proteins in three independent experiments upon immunoprecipitation of SYT1-GFP or free GFP followed by LC-MS/MS analysis.

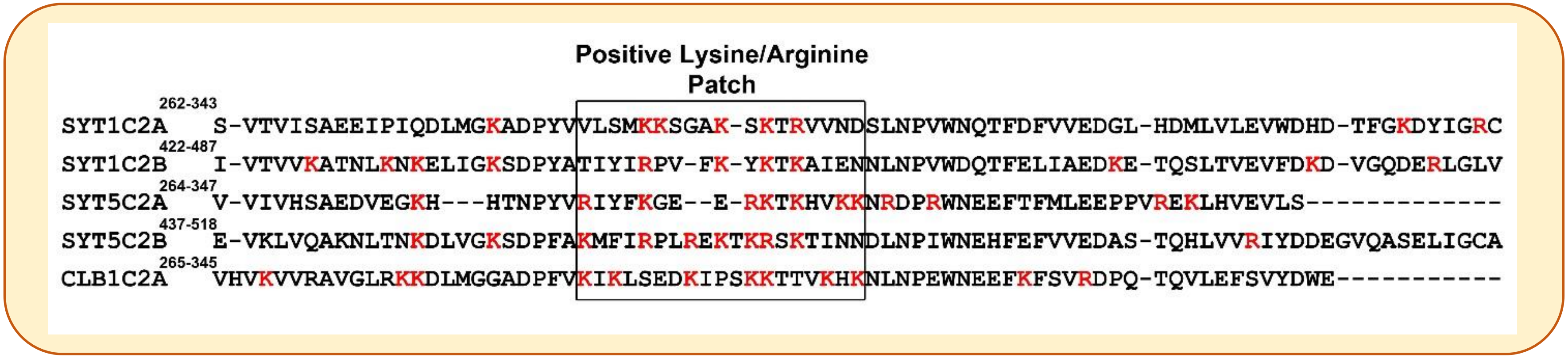

Figure S1. Multiple sequence alignment of the SYT1/SYT5/CLB1 C2 domains. The identification of the C2 regions was determine using Pfam and the multiple sequence alignment was generated using Clustal Omega https://www.ebi.ac.uk/Tools/msa/clustalo/ using seeded guide trees and profile hidden Markov models. The positively charged lysine (K) and arginine (R) aminoacid residues are highlighted in red. The approximate position of the positively charged aminoacid patch is indicated with a rectangle.

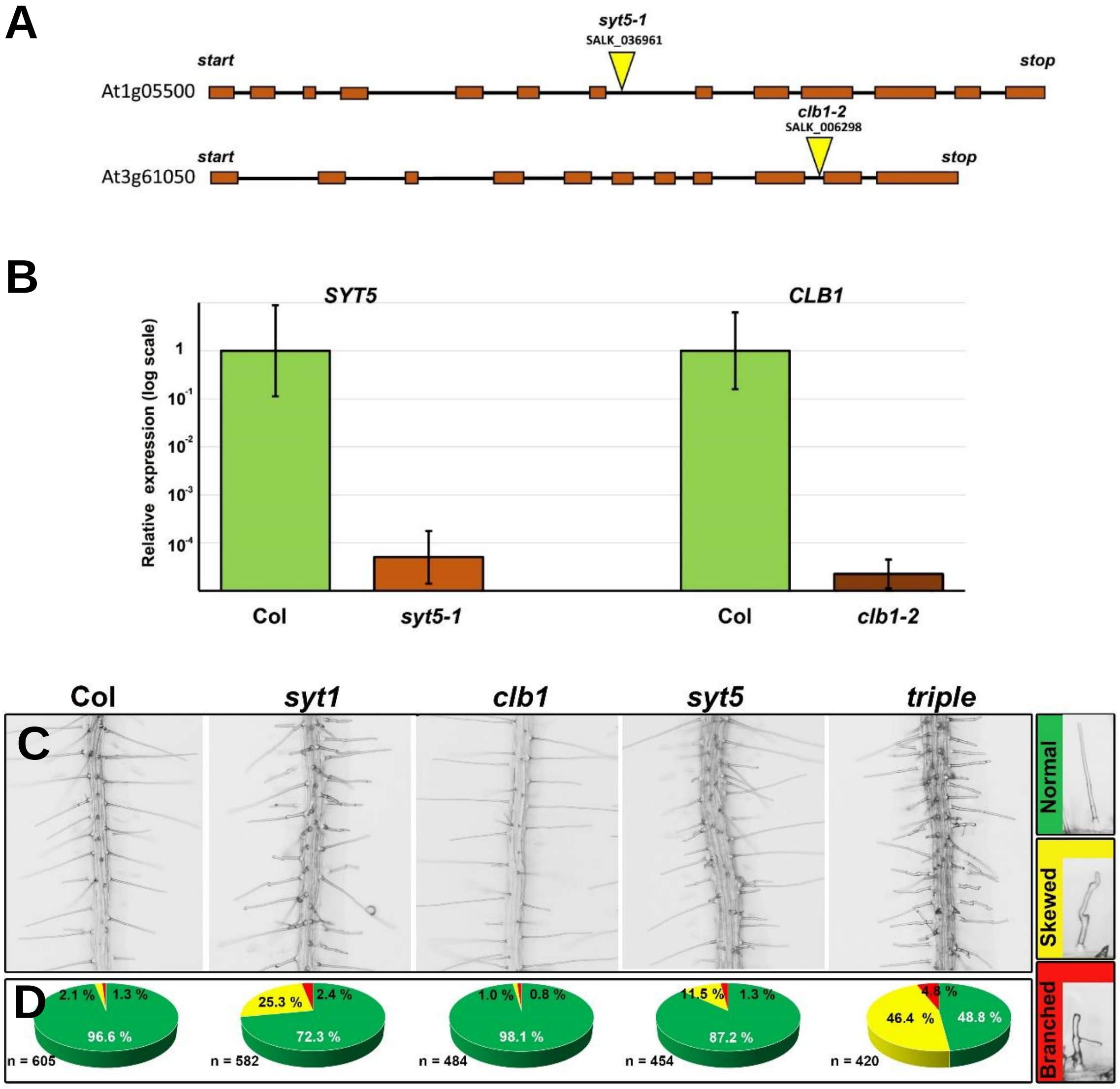

Figure S2. The triple *syt1/syt5/clb1* mutant display root hair polarization defects in the presence of NaCl. A) Exon (orange boxes) – Intron (Black connectors) structured of the At1g05500 and At3g61050 genes. The yellow triangle marks the localization of the T-DNA insertion in the genes. **B)** The graph shows the transcript abundance of SYT5 in WT (Col) and *syt5-1* mutant alleles and CLB1 in WT and *clb1-2* mutant alleles measured by qRT-PCR. The levels of expression for SYT5 and CLB1 in mutant backgrounds are reduced more than 10000 fold compared to their controls **(C)** Representative roots from WT (Col), *syt1-2*, *syt5-1, clb1-2* single and *syt1-2 syt5-1 clb1-2* triple mutants showing the root hair morphology upon NaCl stress. *Arabidopsis* seedlings were grown vertically for 5 d on one tenth-strength MS medium supplemented with 50 mM NaCl and directly imaged on the growth media without mounting. **(D)** Quantification of root hair phenotypes. Right panels show representative root hair morphologies categorized as normal (green), skewed (yellow) or branched (red). Data represent the percentage of root hairs on each category and n represents the number of root hairs quantified for each genotype. Scale bars = 200 µm

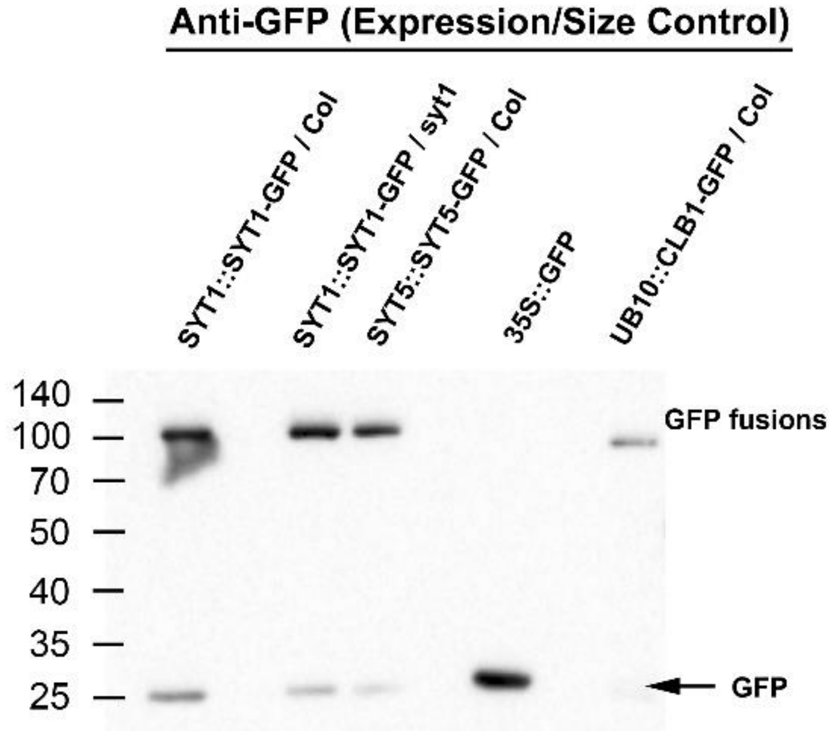

Figure S3. Expression control for immunoprecipitation. Arabidopsis transgenic plants expressing SYT1-GFP, SYT5-GFP and CLB1-GFP were used for immunoprecipitation using GFP-trap beads. Plants expressing free GFP were used as control. The immunoprecipitated proteins were separated by SDS-PAGE (Figure 2), and Western blots were analysed using commercial anti-GFP antibodies as expression control. Molecular weight (kDa) marker bands are indicated for reference.

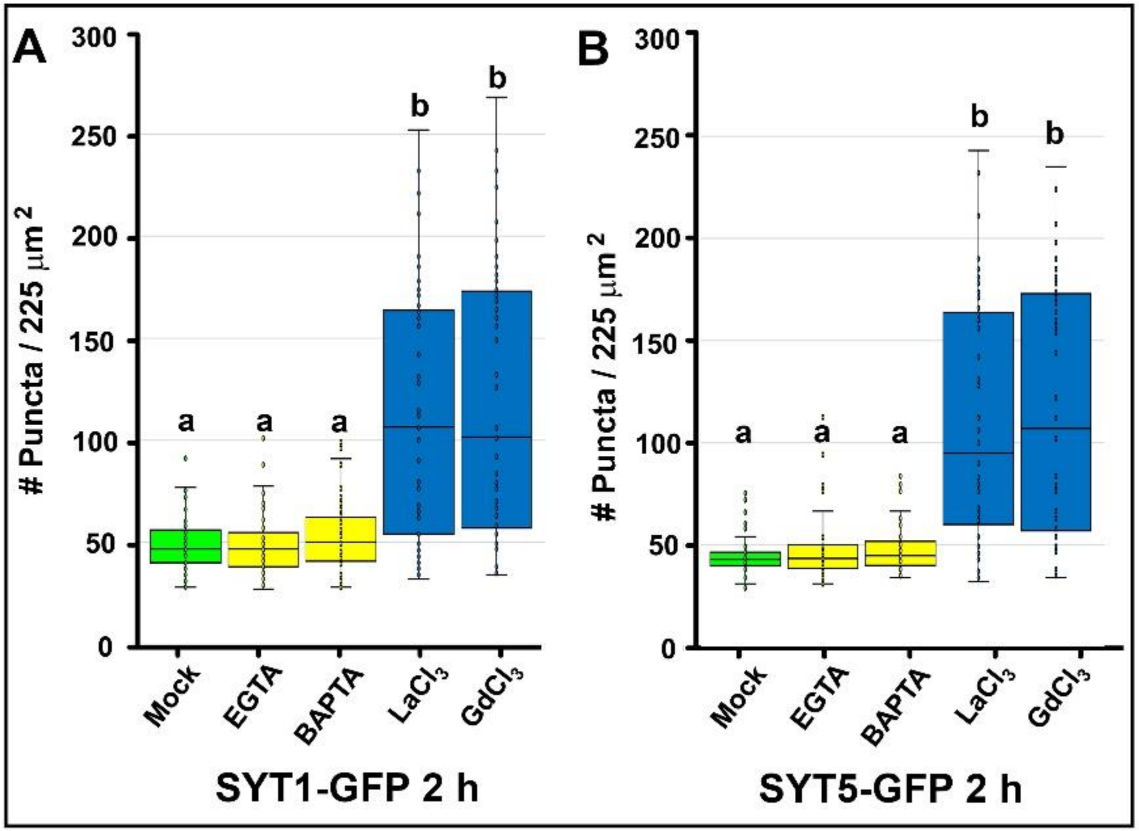

Figure S4. Effect of short-term (2 h) extracellular Ca^{2+} depletion and REE treatments in EPCS number. 5-d-old SYT1-GFP **(A)** or SYT5-GFP **(B)** seedlings were treated in liquid 1/10 strength MS medium supplemented for 2 h with Mock**,** EGTA (5 mM)**,** BAPTA (250 µM)**,** $LaCl_3$ (500 µM), or $GdCl_3$ (500 µM) prior to quantification. The number of SYT1-GFP and SYT5-GFP puncta were scored from 50-60 arbitrary 225 μm^2 ROIs from at least 15 cells from 5 different seedlings. Different letters indicate significant differences among treatments using Tukey multiple pairwise-comparisons p <0.05.

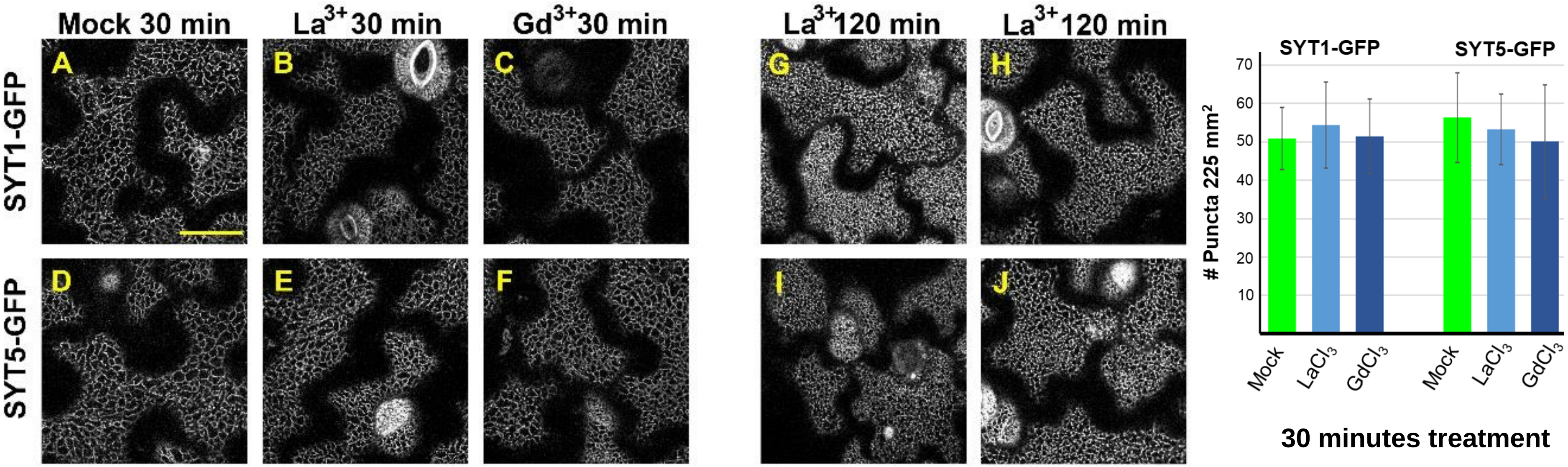

Figure S5. Short-term (30 minutes) REE treatments does not change the EPCS number and overall SYT1-GFP and SYT5-GFP cortical distribution. 5-d-old SYT1-GFP **(A-C)** or SYT5-GFP **(D-F)** seedlings were treated in liquid 1/10 strength MS medium supplemented with Mock (A and D) $LaCl_3$ (500 µM) (B and E) or $GdCl_3$ (500 µM) (C and F) for 30 minutes prior to imaging and quantification. In these experiments a 2 h time point was used as a positive control of REE activity (G-I). (K) EPCS quantification. The number of SYT1-GFP and SYT5-GFP puncta were scored from 20 arbitrary 225 μm^2 ROIs from at least 10 cells from 5 different seedlings. Results are presented as means ± standard deviation (SD). No significant differences among treatments were found using Tukey multiple pairwise-comparisons $p < 0.05$. Scale bars = 20 μm.

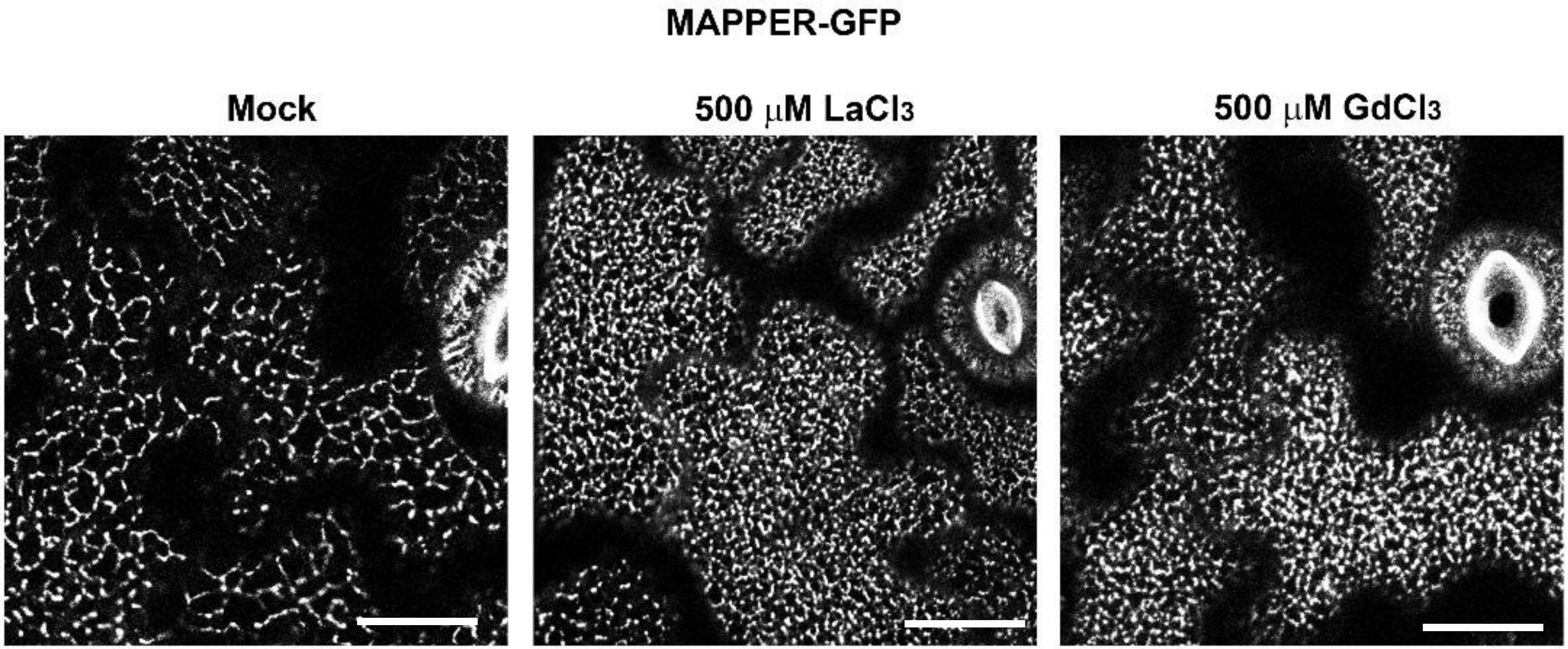

Figure S6. Treatments with non-selective Ca^{2+} channel blockers increase the number of EPCS at the cell cortex. 5-d-old MAPPER-GFP seedlings were treated in liquid one-tenth-strength MS medium supplemented with Mock **(A)** $LaCl_3$ (500 µM / 16 h) **(B)** or $GdCl_3$ (500 µM / 16 h) **(C)** before imaging. EPCS accumulation is indicated by the increased number of MAPPER-GFP puncta at the cell cortex upon REE treatment. Scale bars = 20 µm.

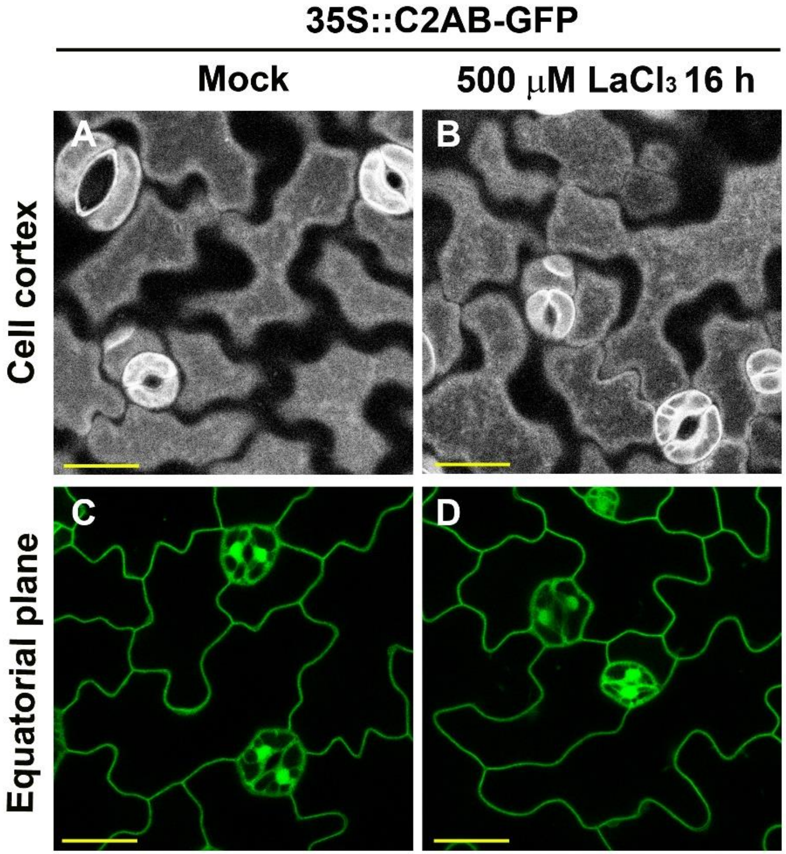

Figure S7. La^{3+} treatments does not induce major changes in the localization of the PM marker C2AB-GFP. 5-d-old 35S::C2AB-GFP seedlings were treated in liquid one-tenth-strength MS medium supplemented with Mock **(A and C)** or $LaCl_3$ (500 µM / 16 h) **(B and D)** before imaging. The images show representative effects of the treatment in the C2AB-GFP subcellular localization at the cell cortex and equatorial planes respectively. Scale bars = 20 µm.

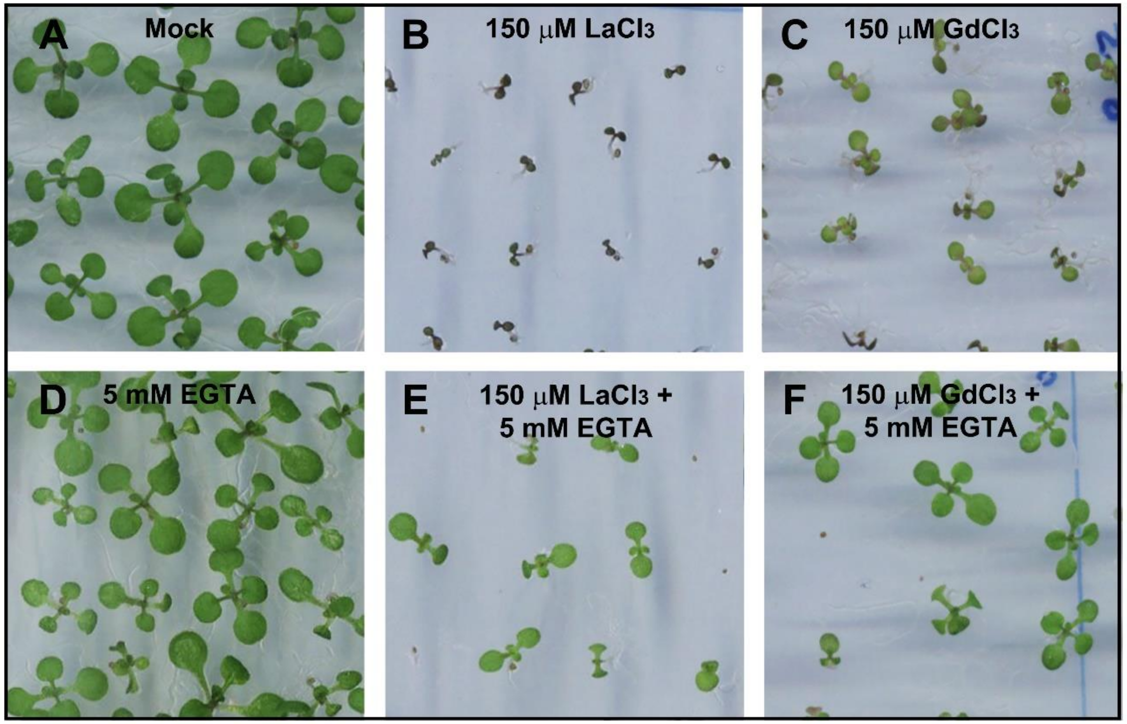

Figure S8. EGTA supplementation partially reverses the growth defects in long-term (14 d) REE-treated seedlings. The images show the development of 14-d-old seedlings grown on one-tenth MS media **(A)** or the same media supplemented with 5mM EGTA **(B)**, 150 μM $LaCl_3$ **(C)**, 150 μM $LaCl_3$ + 5 mM EGTA **D)**, 150 μM $GdCl_3$ **(E)**, or 150 μM $GdCl_3$ + 5 mM EGTA **(F)**.

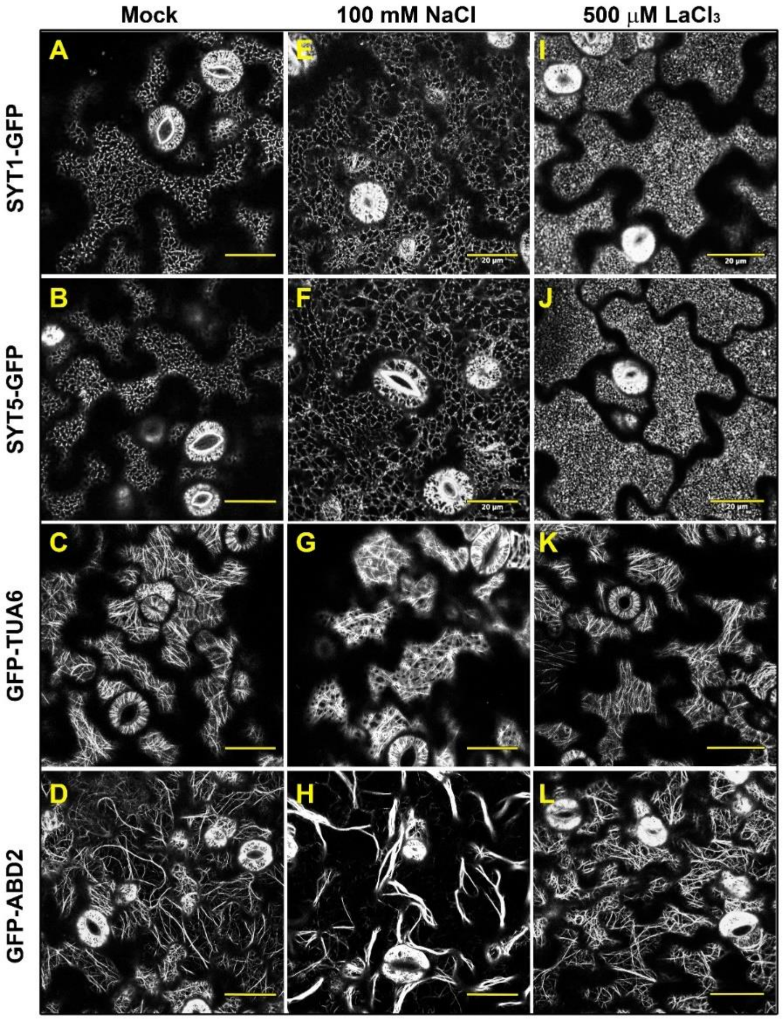

Figure S9. Effect of NaCl and $LaCl_3$ treatments on SYT1-GFP and SYT5-GFP relocalization and cortical cytoskeleton organization. Representative images of the SYT1-GFP and SYT5-GFP S-EPCS markers, GFP-TUA6 microtubule marker, and GFP-ABD2 actin filaments marker upon treatments with mock (A-D), 100 mM NaCl, 16 h (E-H) or 500 µM $LaCl_3$, 16 h (I-L). The images were taken in 5-d-old cotyledon epidermal cells. The effect of the 100 mM NaCl stress was characterized by SYT1-GFP and SYT5-GFP signal expansion along cortical ER tubules, microtubule depolymerization, and actin filaments bundling. The effect of $LaCl_3$ was characterized by an increase in the number of cortical SYT1-GFP and SYT5-GFP structures without gross effects in the cortical microtubules or actin filaments organization. Scale bars (A-L) = 20 µm.

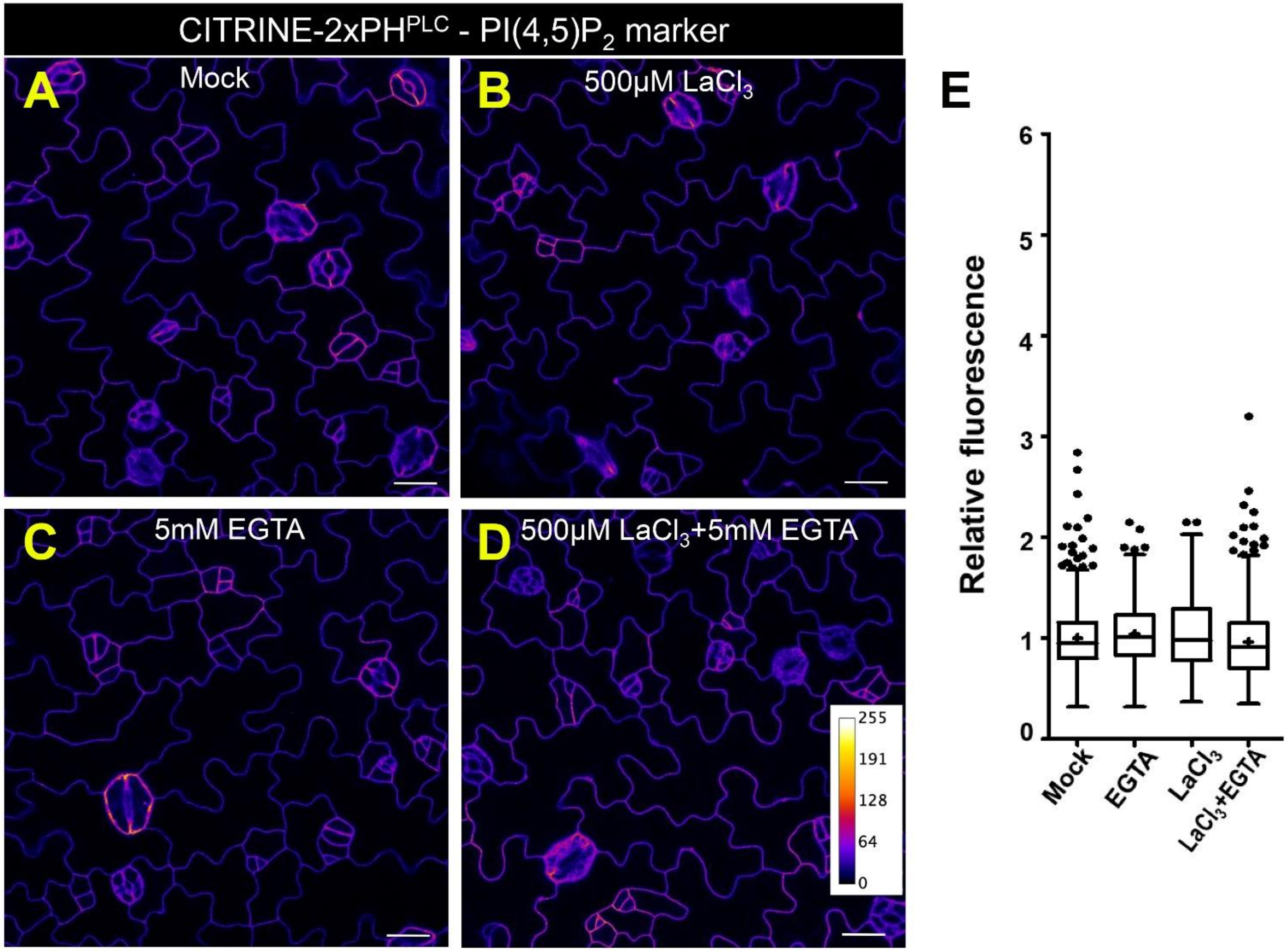

Figure S10. La^{3+} does not induce $PI(4,5)P_2$ accumulation at the PM. (A-D) Confocal images of equatorial planes of cotyledon epidermal cells expressing the CITRINE-2xPH^{PLC} marker. 5-d-old seedlings were treated in liquid one-tenth-strength MS medium supplemented with Mock, 16 h **(A)**, $LaCl_3$ (500 µM / 16 h) **(B)**, EGTA (5 mM / 16h) or $LaCl_3$ (500 µM) + EGTA (5 mM) /16 h **(D)**. $PI(4,5)P_2$ accumulation is shown as color-coded pixel intensity following the LUT scale shown in D. **E)** Quantification of the CITRINE-2xPH^{PLC} signal relative to mock conditions. In the box plots, the center line represents the median fluorescence intensity fold increase relative to mock, the cross represents the mean fluorescent intensity, the top and bottom edges are the 25th and 75th percentiles of the distribution, and the ends of the whiskers are set at 1.5 times the interquartile range (IQR). All values outside the IQR are shown as outliers. At least 100 regions of interest (ROIs) were measured for each treatment. No significant differences among treatments were found using Tukey multiple pairwise-comparisons $p < 0.05$. Scale bars = 20 µm.

Table S2. Primers used in this study

Primer ID	Sequence (5’ to 3’)
CLB1_CDS F	CACCATGGGTTTGATTTCTGGGATTCTG
CLB1_CDS R	CTGCTGTTTTGCACCATCGTTTTCTGG
CLB1_ PROM	CACCCTAATATTATGCACGCTT
SYT5_CDS_F	GGGGACAAGTTTGTACAAAAAAGCAGGCTCCATGGGTTTCATAGTCGGCGTTGTAATCG
SYT5_CDS_R	GGGGACCACTTTGTACAAGAAAGCTGGGTCGGAATCACGATAAATTGATTGAGC
SYT5_PROM	CACCAAGAAAGCGTGATGGCAAAGCCA
